# Predictors on Delay of Initial Health-Seeking in New Pulmonary Tuberculosis Cases among Migrants Population in East China

**DOI:** 10.1371/journal.pone.0031995

**Published:** 2012-02-22

**Authors:** Xinxu Li, Shiwen Jiang, Xue Li, Jian Mei, Qiu Zhong, Weiguo Xu, Jun Li, Weibin Li, Xiaoqiu Liu, Hui Zhang, Lixia Wang

**Affiliations:** 1 National Center for Tuberculosis Control and Prevention, Chinese Center for Disease Control and Prevention, Beijing, People's Republic of China; 2 Shanghai Municipal Center for Disease Control and Prevention, Shanghai, People's Republic of China; 3 Tuberculosis Research Institute of Guangdong Province, Guangzhou, People's Republic of China; 4 Jiangsu Provincial Center for Disease Control and Prevention, Nanjing, People's Republic of China; McGill University, Canada

## Abstract

**Objectives:**

To determine the length of delay in initial health-seeking in new pulmonary tuberculosis (PTB) cases among migrant population in the eastern part of China, and factors associated with it.

**Methods:**

A cross-sectional study was conducted using a structured questionnaire in six counties in Shanghai, Guangdong and Jiangsu from May to October, 2008, to estimate the extent and factors responsible for delayed initial health-seeking of the new PTB cases. The interval between self-reported onset of TB symptoms and date of first attendance at any medical institution was determined. More than the median duration was defined as delayed health-seeking.

**Results:**

A total of 323 new migrant PTB patients participated in the study. Only 6.5% had medical insurance. The median and mean durations to initial health-seeking were respectively 10 and 31 days. There was no significant association between socio-demographic factors and delayed initial health-seeking. Average monthly working days >24 (AOR, 1.61; 95% CI, 1.03–2.51), and hemoptysis or bloody sputum (AOR, 0.48; 95% CI, 0.28–0.85) were significantly associated with delayed initial health-seeking.

**Conclusions:**

Interventions to improve health seeking behavior among the migrant population in China must focus on strengthening their labor, medical security and health education.

## Introduction

From the year 2000 to 2010, the prevalence of any tuberculosis (TB) among the population aged 15 and above changed little from 466 to 459 per 100,000 population in China, yet that for sputum smear-positive TB declined from 169 to 66 per 100,000 population [1]. Nevertheless, because of its sheer population size, China ranked second among the global high TB burden countries in 2009 [2]. Therefore, the challenges ahead in TB prevention and control in China still remain important.

With the rapid development of industrialization and urbanization, China has entered an unprecedented period of population movement and migration. According to the China's Migrant Population Development Report in 2010, there were about 211 million migrants in China by 2009 [3]. Population movements are mainly from the middle and western parts to the eastern part. Approximately 51.6% of the migrant population in China is working in the eastern part [4]. The potential of migration to cause substantial difficulties in the implementation of the National Tuberculosis Program (NTP) becomes apparent from these figures. The prevalence of communicable TB in the western region was the 1.7 and 2.4 times larger than that in the middle and eastern regions respectively [1]. This suggests that migration from the middle and western part may cause an increase in the prevalence in the eastern part and thus conceal any impact the NTP may have. For nationwide control, China must meet the challenges imposed by population migration.

The prevalence survey suggested that only 47% of pulmonary TB (PTB) patients with symptoms sought medical attention [1]. Recent research in China showed that 30.4% of PTB patients had first symptom appearance more than 3 weeks prior to first consultation [5]. Similar observations about delayed diagnosis or delayed treatment have also been made in other countries. For instance, the median interval to diagnosis of infectious TB was 4 weeks in Singapore [6]. In Indonesia, the median total delay for PTB patients in urban districts was 8 weeks compared to 12 weeks for patients in rural districts [7]. Delay in seeking care also exists among the migrant population in China.

Those who have developed symptoms of active PTB, and are in the process of reaching the correct diagnosis and be put on adequate treatment, constitute the bulk of community transmission [8], [9]. Although delayed initial health-seeking covers only a part of the total delay patients, it can impact on transmission. In order to effectively mobilize the migrant population to seek timely medical care, there is a need to better understand their health-seeking behavior and the factors that influence it.

According to official report, in 2009, migrants averagely were 27.3 years old, and those who were 16–59 years old averagely had 9.9 years of education. Approximately 70% of migrants worked in manufacturing, wholesale and retail trade and social services. Monthly expense of per capita household was about 155 US dollars, accounting for 66.3% of monthly income of per capita household. Over 84% of migrants rented the house to live and more than 50% of families lived less than 10 square meters per capita [10]. Furthermore, the health care system was often a great barrier for migrants as only 27%–49% were covered by medical insurance [3], [10]. There might be other barriers to prevent migrant workers to seek help in a timely fashion. It is vital to identify such factors that may cause delayed health-seeking, and to improve TB detection and treatment among the migrant population.

Previous research in Shanghai described the type and length of delay among the migrant population. Income, type of hospital first visiting to, duration in resettlement destination, employment and TB symptoms were associated with the delay of visiting the hospital at the district or higher levels of service [11]. However, lower level health care facilities in China are also responsible for reporting and referring suspected PTB patients to TB dispensaries. The current investigation was designed as a cross-sectional study to investigate demographic characteristics of the migrant population, such as socioeconomic factors, disease manifestations, working and living environment, etc., as potential factors related to delayed initial health-seeking at any health care facility.

## Methods

### Definitions

Migrants were defined as Chinese nationals who retain their registered permanent residence while moving to another area in China [12], where they take up residence or plan to do so for more than 3 months. Excluded from this definition were Chinese people who traveled only, visited relatives or joined the army and those who moved within the same county, same municipal capital, or same provincial capital in China. A new case of PTB from the migrant population was defined as a patient who had been registered and treated for less than 2 months before study begin or who was registered at or after the time this study began.

Duration to initial health-seeking was defined as the interval between date of self-reported symptoms onset and date of first attendance at any health care facility (including hospital, TB dispensary, clinic, health center, etc.). Delayed initial health care-seeking was defined as any duration of delay in excess of the median as ascertained in the study [6]. The self-reported symptoms included major symptoms of PTB, such as cough, expectoration, hemoptysis and bloody sputum, and common symptoms for PTB, such as chest distress, chest pain, mild fever, night sweat, weakness, poor appetite and weight loss [13].

### Participant recruitment

The expected prevalence of delayed initial health care-seeking is 50% using the median as cut-off point [6]. With both type I error rate = 5% and relative error rate = 10–15%, using sample size estimation methods of cross-sectional study, the estimated range of sample size required was approximately 200 to 400 patients. Survey sites were sampled randomly from those counties in Shanghai, Guangdong and Jiangsu, whose number of migrants exceeded 300,000 in 2006. We sampled 2 counties as survey sites per province, for a total of 6 counties in the three provinces split according to the maximum sample size.

The study intake period was uniformly defined as May to October 2008 in the TB dispensaries of all 6 counties. After a diagnosis of PTB patients according to NTP criteria [13], study participants were assessed to determine whether or not they were eligible according to the study enrollment criteria, which included over 18 years old, migrant and new PTB. All enrolled participants signed informed consent papers. The study was approved by the institutional review board (IRB) of the Chinese Center for Disease Control and Prevention.

### Data collection

Questionnaire-based interviews were conducted in the clinic rooms in the county TB dispensaries. Data collected included socio-demographic information (i.e., age, gender, education, employment, marital status, income and expenditure), working hours per day and working days per month, living circumstances (i.e., duration of living in the place, type of accommodation, distance between residence and TB dispensary/hospital), payment method for medical expenses, and information about reasons for medical care-seeking (i.e., main TB symptoms, date of first symptom appearance, health care facility first approached, date of first attendance).

The completed questionnaires were collected by field investigators and were checked for completeness by field supervisors. If a questionnaire was identified to be incomplete, the field investigator was required to conduct a second interview as soon as possible. EpiData software (EpiData 3.1 for Windows™, freely available from the EpiData Association Odense, Denmark, http://www.epidata.dk) was utilized for electronic data capturing from written questionnaires.

### Statistical analysis

The Statistical Analysis System (SAS 9.1 for Windows™, SAS Institute Inc., Cary, NC, USA) was used for data analysis. Descriptive summary measures of frequency and central tendency of the study variables were computed as required. Enrolled participants were divided into 2 groups: delayed initial health-seeking and non-delayed initial health seeking, dichotomized based on the median determined by the data. In univariate analysis, the Chi-square test was used to identify the associations between the study variables and initial health-seeking delay. Multivariate logistic regression, which included all variables with *P* value<0.05 in univariate analysis, was subsequently employed to determine the associations of those factors found to be statistically significant by Chi-square testing.

## Results

A total of 323 participants were enrolled in 3 provinces, including 31.6% in Shanghai, 38.4% in Jiangsu and 30.0% in Guangdong. Except for 5.9% whose initial health-seeking facilities were TB dispensaries, those whose initial health-seeking facilities were general hospitals (42.4%), health centers (27.2%), special hospitals (15.2%) and private clinics (9.3%) were reported and referred to TB dispensaries.

The characteristics of participants are summarized in Table 1. The majority of patients (63.8%) were male. The median age was 30 (interquartile range [IQR] 24–38) years old. In terms of education, 21.4% of participants had elementary school or less and 46.1% had junior high school. The majority of patients (64.1%) were married, and 83.3% of participants had resided for more than 1 year. The median distance between residence and TB dispensary/hospital was 15 (IQR 5–25) kilometers.

**Table 1:** Characteristics of new PTB cases among the migrant population recruited in Shanghai, Guangdong and Jiangsu, China, in 2008.

Variables	No.	%
Gender		
Male	206	63.8
Female	117	36.2
Age (years)*	30 (24, 38)	
Education		
Elementary school or less	69	21.4
Junior high school	149	46.1
Senior high school or higher	105	32.5
Marital status		
Single	106	32.8
Married	207	64.1
Occupation		
Mmigrant workers	194	60.0
Unemployment	65	20.1
Average working hours per day*	8 (6, 10)	
Average monthly working days*	25 (20, 28)	
Type of living quarters		
Rental Housing	237	73.4
Dormitory	49	15.2
Years of living in accommodation place		
≤1	54	16.7
>1	269	83.3
Main source of family income		
Own and other family members' income	158	48.9
Own income	108	33.4
Only other family members' income	57	17.6
The ratio of household expenditure to income in 2007*	0.75 (0.53–0.92)	
Symptoms before initial health-seeking		
cough or expectoration	213	65.9
hemoptysis or bloody sputum	68	21.0
chest distress or chest pain	137	42.4
Fever	84	26.0
No any medical insurance in city	302	93.5
Distances (kilometers) between residence and TB dispensary/hospital*	15 (5, 25)	
Duration (days) of initial health-seeking*	10 (1, 30)	

* Median (1st quartile, 3rd quartile).

Most participants (60.0%) were migrant workers; however, 20.1% were unemployed. Participants worked a median of 8 (IQR 6–10) hours per day and 25 (IQR 20–28) days per month. In 2007, the median total ratio of household expenditure to income was 0.75 (IQR 0.53–0.92). The large majority (93.5%) had no medical insurance in cities and had to pay their medical expenses from their own pocket.

Cough or expectoration (65.9%), and chest distress or chest pain (42.4%) were the most common TB symptoms of participants before initial health-seeking. Of all participants, 43.6% were sputum smear positive and 56.4% were sputum smear negative.

**Figure 1:**
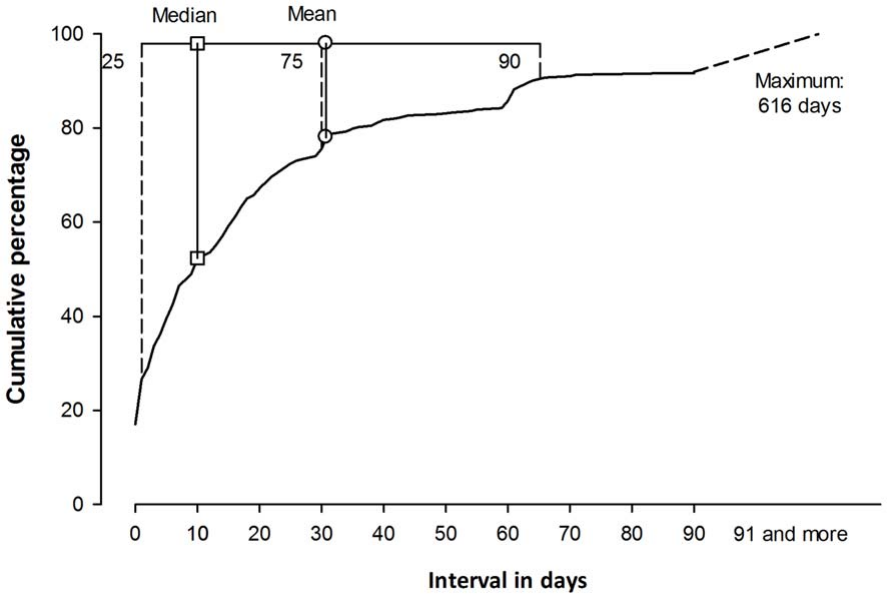
Cumulative interval in days between self-reported symptom onset and attending first health care facility, of new PTB cases among the migrant population recruited in Shanghai, Guangdong, and Jiangsu, China, 2008. The thin horizontal line denotes the range from the 25^th^ to the 90^th^ percentile with the square symbol denoting the median and the circle the mean. Dashed vertical lines are the 25^th^, 75^th^, and 90^th^ percentiles, and the two thin vertical lines the median and mean respectively.

Figure 1 shows the cumulative percentage of interval between self-reported symptoms onset and attendance at the first health care facility. While the median interval was just 10 days, the distribution was much skewed, resulting in a mean of 30.7 days, almost identical with the 75^th^ percentile, and a maximum of more than 1.5 years in one patient. Ninety-two per cent of patients attended within three months (90 days).

Table 2 summarizes the results of the univariate analysis. Delayed initial health-seeking was defined as duration in excess of the median duration (10 days). A total of 167 patients had delayed initial health-seeking by this definition and their characteristics were compared with 154 ones who did not. By univariate analysis, characteristics associated with delayed initial health-seeking were education level being junior high school or less (OR, 2.58; 95% CI, 1.01–6.77), average monthly working days >24 (OR, 1.61; 95% CI, 1.04–2.51), and having hemoptysis or bloody sputum before initial health-seeking (OR, 0.48; 95% CI, 0.28–0.85).

**Table 2:** Univariate analysis of characteristics of new PTB cases among the migrant population and their association with delayed initial health-seeking (IHS).

Variables	≤10 days of delayed IHS (N = 169)	>10 days of delayed IHS (N = 154)	Crude odds ratio (95% CI)	*P* value
	No. (%)	No. (%)		
Gender (Female)	55 (32.5)	62 (40.3)	1.4 (0.9–2.2)	0.1496
Age >30 years	75 (44.4)	76 (49.3)	1.2 (0.8–1.9)	0.3711
Junior high school or less	153 (90.5)	148 (96.1)	2.6 (1.0–6.8)	0.0471
Married	105 (62.1)	102 (66.2)	1.2 (0.8–1.9)	0.4426
Migrant workers	106 (62.7)	88 (57.1)	0.8 (0.5–1.2)	0.3065
Average working hours per day >8	54 (32.0)	57 (37.0)	1.2 (0.8–2.0)	0.3389
Average monthly working days >24	81 (47.9)	92 (59.7)	1.6 (1.0–2.5)	0.0335
Residing in the work shed/dormitory	31 (18.3)	24 (15.6)	0.8 (0.4–1.5)	0.5100
≤1 years of living in accommodation place	27 (16.0)	27 (17.5)	1.1 (0.6–2.0)	0.7081
Responsible for main source of family income oneself	49 (29.0)	57 (37.0)	1.4 (0.9–2.3)	0.1253
The ratio of household expenditure to income in 2007 >0.75	66 (39.0)	56 (36.4)	0.9 (0.6–1.4)	0.6185
Having hemoptysis or bloody sputum before initial health-seeking	45 (26.6)	23 (14.9)	0.5 (0.3–0.8)	0.0100
No any medical insurance in city	156 (92.3)	146 (94.8)	1.5 (0.6–3.8)	0.3632
>15 kilometers between residence and TB dispensary/hospital	72 (42.6)	71 (46.1)	1.2 (0.7–1.8)	0.5270
Comprehensive understanding of national policy for free TB diagnosis and treatment	134 (79.3)	118 (76.6)	0.8 (0.5–1.4)	0.5633
Only willing to let family know after suffering from TB	89 (52.7)	71 (46.1)	0.8 (0.5–1.2)	0.2390
Learning about some knowledge of TB control and prevention	73 (43.2)	56 (36.4)	0.8 (0.5–1.2)	0.2105

Age, gender, employment, marital status, income and expenditure, living circumstances (i.e., living time, type of living quarters, distance between residence and TB dispensary/hospital), medical support status (i.e., payment methods of medical expense) and knowledge, attitude and behavior about TB, were not associated with delayed initial health-seeking.

The results of multivariate logistic regression are shown in Table 3. Average monthly working days >24 (OR, 1.61; 95% CI, 1.03–2.51), and having hemoptysis or bloody sputum before initial health-seeking (OR, 0.48; 95% CI, 0.28–0.85) were significantly associated with delayed initial health-seeking.

**Table 3:** Multivariate analysis of factors associated with delayed IHS.

Covariate	Adjusted odds ration	95% CI	*P* value
Average monthly working days >24	1.6	1.0–2.5	0.0369
Having hemoptysis or bloody sputum before initial health-seeking	0.5	0.3–0.8	0.0118

## Discussion

The findings of this study highlight that the median duration of initial health-seeking of new PTB cases among the migrant population in the eastern part of China was just 10 days. This is half of what was reported from a study conducted in Shanghai, where, among non-resident people, it was 21 days [14]. The endpoint of interval was first attendance at any health care facility in this study but first visit in a district or higher level hospital in the Shanghai study [14], which might be the main reason why the interval identified in our study was fairly short. While a median of ten days appears *prima facie* as a very short interval, yet it is disquieting that half of patients had actually long intervals and only 75% attended within 3 months after symptom onset.

A systematic review examined almost 60 studies on TB patient's and health care system delay and showed the various definitions that have applied [15]. Among the 39 studies that tried to assess similarly to ours the shortest interval between symptom onset and first attendance, the median was even shorter than in our study in four studies. While the median is a good measure of central tendency, it hides the fact of the operationally more important long tail of the half of patients who do not act promptly when symptoms of a potentially serious illness become manifest. We focused on defining the shortest possible interval to initial health-seeking because it is the prerequisite of any TB case detection activity within the health care system.

In recent years, the concept of care-seeking delay or patient delay has garnered increasing attention in the world, with numerous reports such as from Ethiopia [16], Iran [17], Malaysia [18], Cameroon [19], South Africa [20], and Nepal [21]. Delays in these studies ranged from 13 to 50 days, longer than in the current study. Observed differences are likely to be multifactorial.

Having hemoptysis or bloody sputum before initial health-seeking is likely a scary experience and it is not surprising that it readily prompted initial health-seeking in the present study. The finding is consistent with that in Huang's and Wang's studies in Shanghai [11], [14], who reported that those with the more severe TB symptoms were seeking health care earlier than those with less severe ones. In contrast with China's studies, the research in Ethiopia showed that delay among both smear positive and negative patients was longer if they had hemoptysis. A reason might be that patients who stayed home until they were alerted by an alarming symptom such as hemoptysis that finally prompted them to seek care [22]. All these results could similarly suggest that migrants know little about TB symptoms and often ignore the light symptoms. So, it is possibly an expression of the fact that people seek generally only care when the balance between severity of the health problem and the costs associated with seeking care tilt in favor of the latter.

Working for more than 24 days per month was a risk factor for delayed initial health-seeking in the present study and we found more than 75% of our study participants had to work more than 20 days per month. Although this factor was not the focus of other studies [5]–[7], [14]–[23], it highlights an important barrier to attain the opportunities for seeking care and detect the TB cases among the migrant population. Furthermore, patients might be unwilling to ask for leave during work out of concerns of being stigmatized or losing the work. Chinese labor law regulates that working time of whole year is 250 days and average working time per month is not more than 21 days, except for weekends and legal festivals [24]. Not adequate rest days indicates legal rights of most migrants were invaded and they had less labor security in China; however, it is a potentially important contributing factor for delayed initial health-seeking.

Severe symptoms and more monthly work days only partially capture the reasons for delayed initial health-seeking. Other studies have reported a multitude of additional 218 factors, such as duration of residence [11], employment [11], income level [11], [14], the kind of health care facility first visiting to [11], [16], [20], self treatment [16], gender [18], [20], being the main income earner [19], the use of traditional medicine [19], the belief that TB is stigmatizing [19], smoking [21], distance from home to health institute [22], and knowledge about TB [22], [23]. All these were variably reported to be associated with patient delay, but were not found in this study. The very similar background (i.e., age, education, living and work experiences) of the participants in this study might explain why many of the factors reported by others were not identifiable in this study.

Currently, when health care policies were developed and implemented in many cities, migrants were excluded from the coverage of the basic medical insurance. Even if some of migrants were covered by medical insurance in cities, the security levels were lower than that of the local residents. Furthermore, since migrants often floated between different cities to find works, which lead to the discontinuous medical insurance, they actually lost the basic health care rights in cities [25]. We found that 93.5% of total participants had no medical insurance in cities, consistent with the even higher proportion of 99.1% reported in a previous study from Shanghai [11], [14], although official report showed only 27%–49% of migrants had medical insurances, which might include that of their hometown [3], [10].

Of considerable importance might be the fact that virtually none of the migrants is covered by medical insurance in cities. Because this status applied to the majority of our study participants, it precluded its identification as a contributing factor. Lack of medical insurance may thus be another important barrier to prevent the migrant population accessing medical care in cities. It nevertheless requires more attention because one may infer that having no medical insurance would almost certainly entail delaying seeking care until health concerns become sufficiently severe. An improvement of TB case detection might remain seriously hampered if this state continues to persist.

Shanghai, Guangdong and Jiangsu lie in the eastern part of China, a well developed area, where the infrastructure of health care system is fairly good and DOTS services are easily accessible for most patients, especially for the migrant population, if they actively seek help from a health care facility. Therefore, according to the result of this study, it should be recognized that the TB case-detection among the migrant population of this area will be improved remarkably as long as their labor and medical security and health education about TB knowledge are strengthened.

The current study has potential limitations. Firstly, selection bias may not have been entirely avoidable because the study was restricted to migrants with PTB and we only enrolled participants who presented at TB dispensaries. This way of recruiting patients for delay studies would miss out general migrants and all those who did not reach a correct diagnosis. As it was not based on strict random sampling, our results cannot necessarily be generalized to all new PTB patients among the migrant population in these regions. Second, recall bias may have occurred due to our outcome measure of delay in initial care-seeking being self-reported. This problem is inherent to all studies of this type as there is no gold standard to define symptom onset and no valid alternative has yet been identified. To reduce the recall bias, questions need to be specially targeted to identify the onset of any, not only of the major pre-occupying TB symptoms.

In conclusion, we have shown that 93.5% of new PTB cases among the migrant population lack medical insurance in this study; the median total duration of initial health-seeking was 10 days, but the mean was 30.7 days and the delay was associated with work days and TB symptoms. Interventions to improve health seeking behavior among the migrant population in China must focus on strengthening their labor and medical security and health education, rather than considering their socio-demography more.
